# Genetic variation, population structure and linkage disequilibrium in Switchgrass with ISSR, SCoT and EST-SSR markers

**DOI:** 10.1186/s41065-016-0007-z

**Published:** 2016-04-19

**Authors:** Yu Zhang, Haidong Yan, Xiaomei Jiang, Xiaoli Wang, Linkai Huang, Bin Xu, Xinquan Zhang, Lexin Zhang

**Affiliations:** 1 Grassland Science Department, Sichuan Agricultural University, Chengdu, 611130 China; 2 IRTA. Centre de Recerca en Agrigenòmica (CSIC-IRTA-UAB), Campus UAB – Edifici CRAG, Bellaterra - Cerdanyola del Vallès, Barcelona, 08193 Spain; 3 Guizhou Institute of Prataculture, Guiyang, 550006 PR China; 4 College of Grassland Science, Nanjing Agricultural University, Nanjing, 210095 China

**Keywords:** Genetic variation, Linkage disequilibrium, *Panicum virgatum* L, Population structure

## Abstract

**Background:**

To evaluate genetic variation, population structure, and the extent of linkage disequilibrium (LD), 134 switchgrass (*Panicum virgatum* L.) samples were analyzed with 51 markers, including 16 ISSRs, 20 SCoTs, and 15 EST-SSRs.

**Results:**

In this study, a high level of genetic variation was observed in the switchgrass samples and they had an average Nei’s gene diversity index (H) of 0.311. A total of 793 bands were obtained, of which 708 (89.28 %) were polymorphic. Using a parameter marker index (MI), the efficiency of the three types of markers (ISSR, SCoT, and EST-SSR) in the study were compared and we found that SCoT had a higher marker efficiency than the other two markers. The 134 switchgrass samples could be divided into two sub-populations based on STRUCTURE, UPGMA clustering, and principal coordinate analyses (PCA), and upland and lowland ecotypes could be separated by UPGMA clustering and PCA analyses. Linkage disequilibrium analysis revealed an average r^2^ of 0.035 across all 51 markers, indicating a trend of higher LD in sub-population 2 than that in sub-population 1 (*P* < 0.01).

**Conclusions:**

The population structure revealed in this study will guide the design of future association studies using these switchgrass samples.

## Background

Genetic diverstiy is a significant factor that contributes to crop improvement. Evaluation of genetic variation in contemporary germplasm through breeding programs may be indirectly favorable for genetic progress in future cultivars [1]. Thus, estimation of plant diversity is crucial for the efficacious use of genetic resources in breeding programs. Molecular markers, as particular segments of DNA that represent different functional classes, play an essential role in all aspects of plant breeding, and have been widely used to estimate genetic variation.

Compared with conventional phenotyping methods, molecular markers have numerous advantages as they are easily detectable and stable in plant tissues regardless of environmental influences [2]. The inter simple sequence repeat marker (ISSR) is highly polymorphic and is useful in studies of genetic diversity, genome mapping and evolutionary biology [3]. This PCR-based technique is used in various types of plants and can overcome many defects of other marker methods, such as high-cost of amplified fragment length (AFLP) and the low reproducibility of random amplified polymorphic DNA (RAPD) [4]. Start codon targeted marker (SCoT) is a reliable and simple gene-targeted marker located on the translational start codon [5]. This technique involves designing single primers from the short conserved region flanking the ATG start codon [6] without knowing any further genomic sequence information. It has been used in peanut and mango crops for genetic diversity and cultivar relationship analysis [7]. Expressed sequence tag-simple sequence repeats marker (EST-SSR) detects variation based on the expressed portion of the genome from EST databases, thus explaining the low cost of development compared with the genomic simple sequence repeat marker (SSR) [8]. These EST-SSR primers can be used across various species for comparative mapping and the construction of genetic linkage maps [9, 10]. Each marker type has unique advantages and these three marker systems have found extensive application in the evaluation of genetic variation, population structure, and assisted selection for crop improvement [3, 11–14]. Many studies have shown that these markers are mainly used to develop genetic linkage maps [15, 16], however, fewer studies have focused on constructing linkage disequilibrium (LD) maps. Remarkably, LD and linkage are two different genetic terms, where LD refers to correlation between alleles in a population, while LD means the correlated inheritance of loci through physical connection on a chromosome [17]. Some factors can affect the LD level, including allele frequency and recombination. Unlike linkage analysis, LD mapping relies on a natural population which is used to identify the relationships between genetic and phenotypic variation. LD mapping, that is association analysis, represents a useful tool to identify trait-marker relationships, and the first LD mapping of a quantitative trait was the analysis of flowering time and the *dwarf8* gene in maize [18].

Linkage disequilibrium (LD), referring to the nonrandom association of alleles between linked or unlinked loci, is the basis of association mapping to identify genetic regions associated with agronomic traits [17]. Recently, LD studies have been performed in various plants, such as rice (*Oryza sativa* L.) [19], barley (*Hordeum vulgare* L.) [20], Maize (*Zea mays* L.) [21], chickpea (*Cicer arietinum* L.) [22], perennial ryegrass (*Lolium perenne* L.) [23], and the model legume, *Medicago truncatula* [24]. The level of LD is constantly regarded as a standard to reflect mapping resolution. Association mapping in populations with low LD requires a high number of markers, whereas a high LD means low mapping resolution [25]. In addition, information about population structure within germplasm collections is also crucial for the interpretation and identification of associations between genetic and functional diversity, and to assess whether the inter-sample relatedness is suitable for association studies [26–28]. Therefore, population structure is also included as an effect in models used for association analysis. [15, 29].

Switchgrass (*Panicum virgatum* L.), as a warm season C_4_ perennial grass that is native to North America [30], is regarded as an important biofuel crop for its remarkable biomass yield and good adaptability on marginal lands thereby not competing with food crops on farmland [31–33]. In this study, we explored two distinct forms of switchgrass, upland and lowland ecotypes. The upland accessions are distributed in northern cold areas with lower biomass than lowland varieties. Generally, upland switchgrass is shorter (≤2.4 m, tall) than lowland types (≥ 2.7 m) in favorable environments. However, lowland cultivars appear more sensitive to moisture stress than upland cultivars [34].

Constructing association maps comparing the physiological and genetic basis of varying stresses can provide an available reference for the genetic improvement of switchgrass, and the evaluation of the level of LD and population structure can aid association analyses. To date, however, LD analysis across the switchgrass genome remains inadequate [35]. In our study, we present 134 switchgrass accessions supplied by Plant Genetic Resources Conservation Unit, Griffin, Georgia USA to identify the levels of genetic variation, population structure, and extent of LD using 51 markers including 16 ISSRs, 20 SCoTs, and 15 EST-SSRs. These results will provide a valuable molecular basis for enriching switchgrass genetic variation, and the information on the level of LD and population structure may guide association mapping using this representative collection.

Here we constructed a three-marker molecular dataset with important applications for diversity analysis, establishment of population structure and evaluation of linkage disequilibrium in switchgrass which is an allogamous species.

## Results and discussion

### Genetic variation analysis

The ISSR, SCoT, and EST-SSR primers were screened using the selected four genotypes [PI421999 (AM-314/MS-155), PI422006 (Alamo), PI642190 (Falcon), and PI642207 (70SG 016)]. After the initial screening, the numbers of selected ISSR, SCoT, and EST-SSR primers used in further studies were reduced to 16, 20, and 15 pairs, respectively (Table 1).

**Table 1:** The ISSR, SCoT, and EST-SSR primers used in this study and amplification results

Primer	Primer sequence (5′ → 3′)	Annealing (°C)	Total number of amplified bands (TNB)	The number of polymorphic bands (NPB)	Percentage of polymorphic bands (PPB) %)
ISSR-UBC812	(GA)_8_A	52.0	13	12	92.31
ISSR-UBC827	(AC)_8_G	53.0	10	10	100.00
ISSR-UBC828	(TG)_8_A	52.0	9	8	88.89
ISSR-UBC829	(TG)_8_C	52.0	12	10	83.33
ISSR-UBC830	(TG)_8_G	55.0	14	13	92.86
ISSR-UBC835	(AG)_8_YC	52.0	15	13	86.67
ISSR-UBC836	(AG)_8_YT	54.0	17	14	82.35
ISSR-UBC844	(CT)_8_RC	52.0	14	12	85.71
ISSR-UBC848	(CA)_8_RG	53.0	14	13	92.86
ISSR-UBC854	(TC)_8_RG	52.0	15	15	100.00
ISSR-UBC868	(GAA)_6_	55.0	13	12	92.31
ISSR-UBC876	(GATA)_2_(GACA)_2_	52.0	16	13	81.25
ISSR-UBC879	(CTTCA)_3_	53.0	17	14	82.35
ISSR-UBC887	DVD(TC)_7_	52.0	14	14	100.00
ISSR-UBC890	VHV(GT)_7_	52.0	13	11	84.62
ISSR-UBC891	HVH(TG)_7_	52.0	14	12	85.71
SCoT2	CAACAATGGCTACCACCC	55.0	17	14	82.35
SCoT3	CAACAATGGCTACCACCG	55.0	34	31	91.18
SCoT4	CAACAATGGCTACCACCT	55.0	21	19	90.48
SCoT5	CAACAATGGCTACCACGA	55.0	21	19	90.48
SCoT6	CAACAATGGCTACCACGC	55.0	22	20	91.91
SCoT7	CAACAATGGCTACCACGG	55.0	22	20	91.91
SCoT9	CAACAATGGCTACCAGCA	55.0	20	19	95.00
SCoT10	CAACAATGGCTACCAGCC	55.0	21	20	95.00
SCoT12	ACGACATGGCGACCAACG	55.0	25	24	96.00
SCoT13	ACGACATGGCGACCATCG	55.0	25	22	88.00
SCoT15	ACGACATGGCGACCGCGA	55.0	18	16	88.89
SCoT16	ACCATGGCTACCACCGAC	55.0	26	24	92.31
SCoT18	ACCATGGCTACCACCGCC	55.0	20	18	90.00
SCoT21	ACGACATGGCGACCCACA	55.0	21	19	90.48
SCoT28	CCATGGCTACCACCGCCA	55.0	25	22	88.00
SCoT31	CCATGGCTACCACCGCCT	55.0	22	19	86.36
SCoT34	ACCATGGCTACCACCGCA	55.0	21	19	90.48
SCoT35	CATGGCTACCACCGGCCC	55.0	21	19	90.48
SCoT37	CAATGGCTACCACTAGCC	55.0	23	20	86.96
SCoT48	ACAATGGCTACCACTGGC	55.0	20	18	90.00
EST-SSR-cnl35	f: AAGTGAGCACAACGACACGA	58.0	9	8	88.89
	r:CGATCCAAAGAAGCAAAGATG				
EST-SSR-cnl37	f:CTGCCTCGCGTGAAAGATA	59.0	10	9	90.00
	r:CCTCCTCGATCTGGATGGT				
EST-SSR-cnl42	f:GTTGGTCTGCTGCTCACTCG	59.0	9	8	88.89
	r:CCGACGATGTTGAAGGAGAG				
EST-SSR-cnl47	f: GACTCGCACGATTTCTCCTC	57.0	9	8	88.89
	r:GCCAGACAACCAATTCAGGT				
EST-SSR-cnl51	f:CTAGGGTTTCCCACCTCTCA	59.0	8	6	75.00
	r:AATGTCCTTGGCGTTGCT				
EST-SSR-cnl55	f:GCTGATAGCGAGGTGGGTAG	58.0	14	11	78.57
	r:CTGCCGGTTGATCTTGTTCT				
EST-SSR-cnl61	f:CACGAGTGCAGAGCTAGACG	60.0	5	4	80.00
	r:ACAACAACCCGACTGCTACC				
EST-SSR-cnl86	f:CAACAACGTCAACGCCTTC	59.0	11	8	72.73
	r:GCGTCTTGAACCTCTTGTCC				
EST-SSR-cnl100	f:CGTCGTCCTCTGCTGTGAG	58.0	5	4	80.00
	r:AGGTCGTCCATCTGCTGCT				
EST-SSR-cnl115	f:CGAGAAGAAGGTGGTGTCGT	59.0	7	6	85.71
	r:AGGTCGTGGAAGGTCTTGG				
EST-SSR-cnl119	f:ATCGTCTCCTCCTCCTCCA	57.0	6	6	100.00
	r:ATGCCTCGGTGGACTGGTA				
EST-SSR-cnl130	f:AAATGTTGAGCAACGGGAGCT	59.0	7	6	85.71
	r:ACTTCATAGGGCGGAGGTCT				
EST-SSR-cnl144	f:AGAAGGCGGCTCAGAAGAAG	58.0	10	10	100.00
	r:GCTCCAACTCAGAATCAACAA				
EST-SSR-cnl147	f:GGCTAGGGTTTCGACTCCTC	60.0	9	7	77.78
	r:AGATGGCGAACTCGACCTG				
EST-SSR-cnl158	f:CTCATCCCACCACCACCAC	59.0	9	9	100.00
	r:CCCTGAAGAAGTCGAACACG				
Total			793	708	89.28

These three marker systems (ISSR, SCoT, and EST-SSR) have been used for cultivar identification and genetic variation assessment in many plant species [36–39]. In this study, these markers were successfully used to differentiate switchgrass accessions. A total of 51 primer pairs were used and 793 bands were produced, with a mean of 15.5 bands per primer, among which 89.28 % were polymorphic. Our results suggested that ISSR, SCoT, and EST-SSR analyses could contribute to the detection of genetic variation. In addition, Nei’s (1973) gene diversity index (H) and Shannon’s information index (I) was 0.311 and 0.471, respectively, and the similarity coefficient, ranging from 0.162 to 0.857 with an average of 0.510 was similar to other studies on switchgrass, in which the similarity coefficients were estimated to be between 0.45 to 0.81 [40] or 0.53 to 0.78 [41]. This indicates that switchgrass has abundant genetic variation and is a highly heterogenous species [42]. The AMOVA of the distance matrix for the genotypes permitted a partitioning of the overall variation into two levels: between upland and lowland ecotypes and within a population. The results revealed genetic differentiation between upland and lowland ecotypes (*P* < 0.001), with 31.42 % of genetic variation between ecotypes and 68.58 % of genetic variation within ecotypes. Similar results were obtained in other switchgrass germplasm collections [40, 43, 44] and in other perennial, and cross-pollinated plants [45].

### Marker efficiency analysis

In this study, we extracted genomic DNA from an individual so that we were able to obtain complete genetic information including allele numbers, gene frequency and observed heterozygosity for marker efficiency analysis. A parameter marker index (MI) was used to compare the efficiencies of the three assays in the collection of 134 switchgrass genotypes (Table 2). There was almost no disparity between the average band informativeness (Ib_av_) indice for ISSRs, SCoTs, and EST-SSRs, which were 0.38, 0.43, and 0.36, respectively. However, the effective multiplex ratio (EMR) index for ScoT (20.10) was twice as high as that of the ISSRs (12.25) and three times as high as that of the EST-SSRs (7.33). The MI calculation indicated an efficient and distinctive nature of the SCoTs with the MI for these markers (8.64) higher than the other two assays examined here (4.66 for ISSRs and 2.64 for EST-SSRs).

**Table 2:** Comparison of usefulness between ISSR, SCoT, and EST-SSR markers for 134 switchgrass accessions

Items	ISSR	SCoT	EST-SSR
No. of primers	16	20	15
No. of total bands	220	445	128
No. of average bands per primers	13.75	22.25	8.53
Percentage of polymorphic bands (PPB)	0.89	0.90	0.86
Average band informativeness (Ib_av_)	0.38	0.43	0.36
Effective multiplex ratio (EMR)	12.25	20.10	7.33
Marker index (MI)	4.66	8.64	2.64

A parameter MI, has been widely used to evaluate the overall utility of each marker system [46]. The high MI in the SCoTs results from its high EMR, making these markers appropriate for fingerprinting [47] or evaluating genetic variation in breeding populations [48, 49]. In addition, the SCoTs performed well in other species. Compared with ISSR and inter-retrotransposon amplified polymorphism (IRAP), SCoT markers were more informative than IRAP and ISSR for the assessment of diversity among Persian oak (*Quercus brantii* Lindl.) individuals [50]. Results from the evaluation on the genetic variation of mango (*Mangifera indica* L.) cultivars indicated that the SCoT analysis represents actual relationships better than the ISSR analysis [51].

### Population structure analysis

**Fig. 1:**
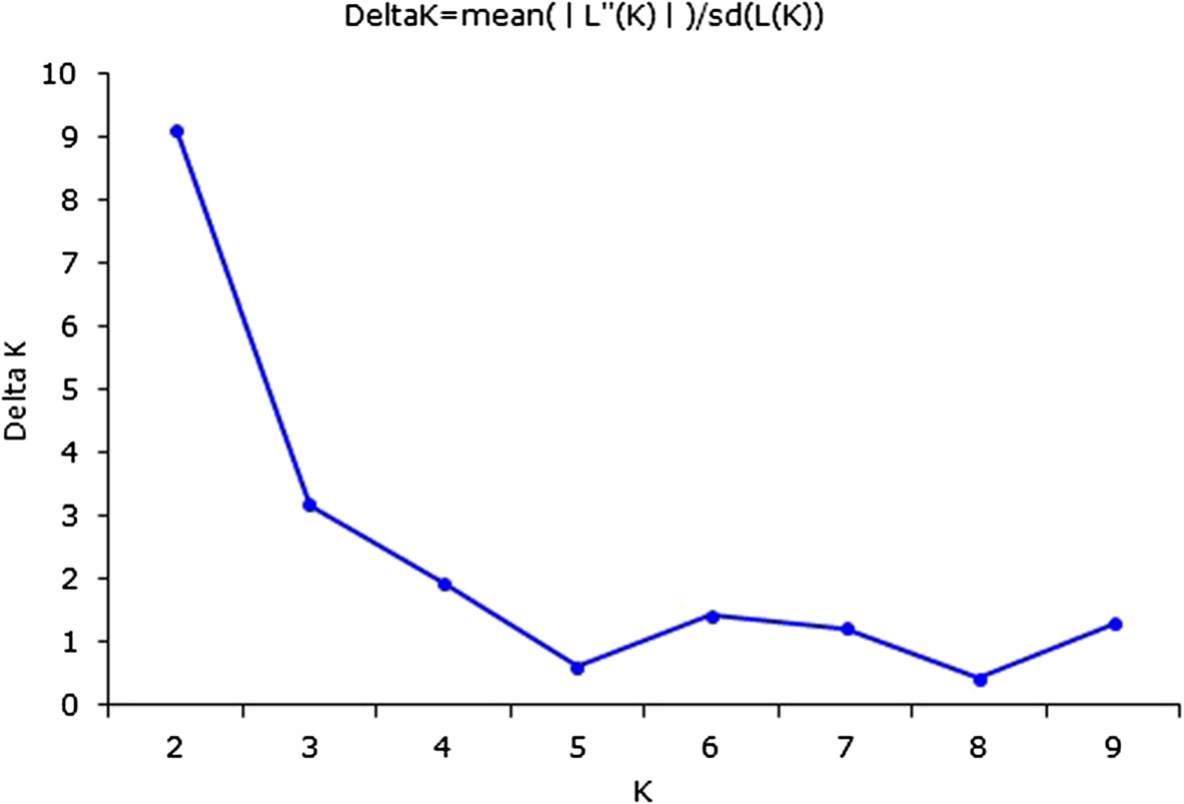
STRUCTURE analysis of the number of populations for K. The number of subpopulations (K) was identified based on maximum likelihood and ΔK values. The most likely value of K identified by STRUCTURE was observed at K = 2

**Fig. 2:**
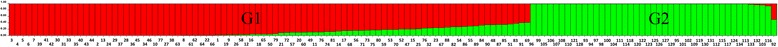
Two subgroups inferred from STRUCTURE analysis. The vertical coordinate of each subgroup means the membership coefficients for each accessions; the digits of the horizontal coordinate represent the 134 switchgrass accessions corresponding to Table 3; Red zone: G1, Green zone: G2

After removing low frequency bands (considering MAF ≤ 0.05), we analyzed the data from 51 pairs of ISSR, SCoT, and EST-SSR primers to understand the population structure of the entire switchgrass collection based on a Bayesian clustering approach using STRUCTURE [52]. The number of subpopulations (K) was identified based on maximum likelihood and ΔK values. For the 134 switchgrass genotypes the maximum ΔK was observed at K = 2 (Fig. 1), with genotypes falling into two subpopulations. Using a membership probability threshold of 0.75, 76 genotypes were assigned to subpopulation 1 (G1), out of which, 69 genotypes belonged to upland ecotypes, and the remaining 7 were lowland. Subpopulation 2 (G2) contained 42 genotypes, and all of them were upland ecotypes. The remaining 16 genotypes were classified into an admixed group as they had membership probabilities lower than 0.75 in any given subpopulation. With the maximum membership probability, 91 accessions were assigned to G1 and 43 accessions to G2 (Fig. 2).

**Fig. 3:**
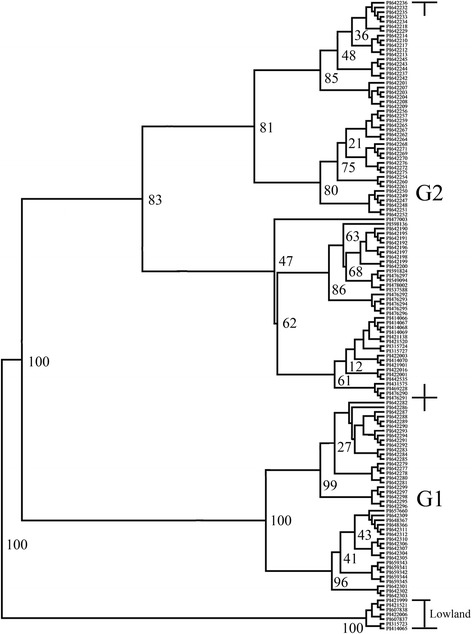
Radiation of genetic relationships for 134 switchgrass accessions based on UPGMA. G1 and G2 are the two subgroups identified by STRUCTURE with the maximum membership probability. The numbers at the branches are confidence values based on Felsenstein’s bootstrap produced by FreeTree software, as a general rule, the higher bootstrap value for a given interior branch indicates a closer relationship

The UPGMA cluster analysis from 51 markers generated a dendrogram, demonstrating that the 134 genotypes could be clearly divided into two groups (Fig. 3). The dendrogram clustered all of the lowland ecotypes (LL) into the first. The second group contained all of the upland ecotypes (UL). Other methods have also been used to cluster upland and lowland switchgrass ecotypes. Missaoui et al adopted restriction fragment length polymorphism (RFLP) markers to analyze the genetic relationships among 21 switchgrass genotypes, resulting in three upland and eighteen lowland genotypes clusteringinto two different groups [53]. Huang et al identified differences between the coding sequences of a nuclear gene encoding plastid acetyl-CoA carboxylase in upland and lowland ecotypes genetic variation analysis at gene level, provided by Huang et al researching about a nuclear gene encoding plastid acetyl-CoA carboxylase [54]. In this study, we preliminarily presented population structure analysis of 7 lowland and 127 upland genotypes using 51 ISSR, SCoT, and EST-SSR primer pairs, resulting in an apparently separate cluster among the two ecotypes, confirming the genetic differences between upland and lowland ecotypes. However, as we do not have as many lowland switchgrass samples as upland, we highly recommend more lowland ecotype or other nuclear markers should be used in conjunction with ISSR, SCoT and EST-SSR to more appropriately classify upland and lowland ecotypes.

**Fig. 4:**
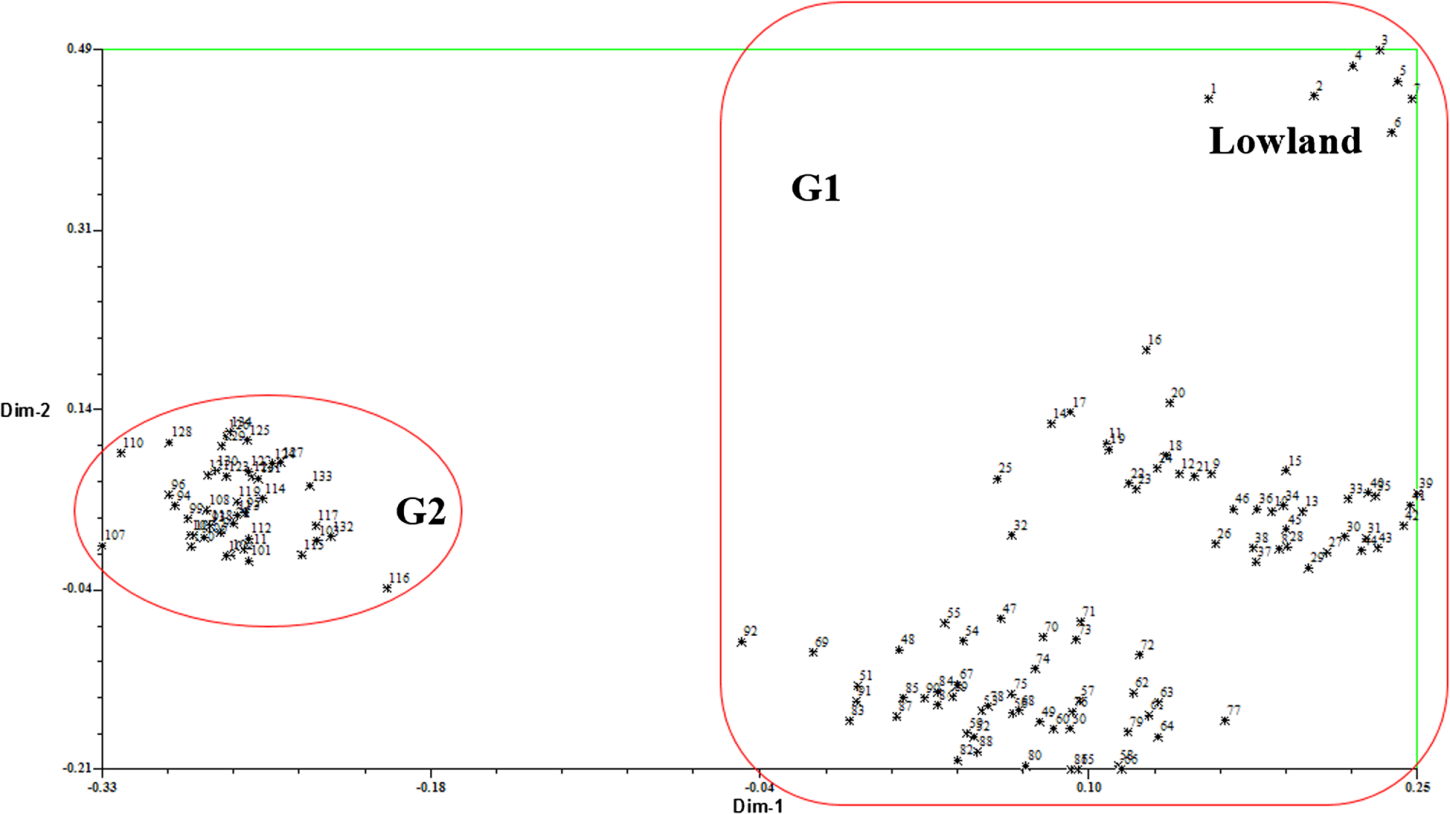
Principal coordinate analysis of 134 switchgrass accessions based on ISSRs, SCoTs, and EST-SSRs. G1 and G2 are the two subgroups identified by STRUCTURE with the maximum membership probability

Based on modified Rogers distances (MRD), PCA separated the 134 genotypes into two major groups, which was consistent with assignments generated by STRUCTURE and the UPGMA dendrogram (Fig. 4). Seven genotypes formed group 1 (Fig. 4, upper right), and the other 127 genotypes, belonging to group 2, were mainly distributed at the lower portion of the plot. The accessions belonging to G1 inferred by the STRUCTURE analysis were all distributed on the right portion of the resulting plot, while G2 was distributed on the left portion of the plot. The distribution of G1 accessions was less tightly clustered than G2, indicating accessions in G1 had higher diversity than G2 (Fig. 4).

Before analyzing LD and association mapping, the analysis of population structure emphasizes the need for the genetic analysis of different ecotypes [28]. The UPGMA cluster and PCA analysis demonstrated that 134 genotypes could be clearly divided into two groups (Figs. 1 and 4), and the lowland and upland germplasm clusters were almost completely separated, which was consistent with the results of several other switchgrass studies [41, 55, 56]. For the UPGMA cluster analysis, the first group only included lowland ecotypes, while the second group contained upland ecotypes and could be further classified into two subgroups. Subgroup 1 (G1) contained 83 genotypes, while the remaining 43 belonged to subgroup 2 (G2). The 46 accessions of the 70SG series and 42 accessions of the 71SG series dispersed into these two subgroups are from the same geographical distribution of North Dakota, United States. This indicates that most of the germplasm sub-clustered in accordance with different regions [43, 55], and the assignment of 132 accessions (98.51 % of the total) by the UPGMA cluster analysis was consistent with their classification using PCA (Fig. 4). Unexpectedly, in the STRUCTURE analysis, the 127 upland genotypes were assigned to two subpopulations, possibly because the UPGMA and STRUCTURE programs calculate parameters in different ways. Clusters are generated in STRUCTURE based on both transitory Hardy–Weinberg disequilibrium and LD caused by admixture between populations [55], while the UPGMA dendrogram generates clusters based on the genetic distance among populations [57].

### Linkage disequilibrium estimation

After the deletion of low frequency alleles (MAF ≤ 5 %), the 51 ISSRs, SCoTs, and EST-SSRs with unknown chromosome information were used to evaluate the extent of LD among the switchgrass samples. In the collection, interallelic r^2^ values, the association between any pair of alleles from different loci, were calculated and ranged from 0.000 to 1.000 with an average r^2^ of 0.035. Across all 51 loci, 247,456 locus pairs were detected in the 134 switchgrass samples. Among all of the locus pairs, 7107 of 135,718 (5.24 %) showed LD at the *P* < 0.001 level for G1 and 5415 locus pairs (3.99 %) were found at r^2^ > 0.1 at *P* < 0.001. For G2, 84,154 locus pairs were detected, 4833 were significant pairs (*P* < 0.001, 5.74 %), while 4235 locus pairs (5.03 % of 84,154) were found at r^2^ > 0.1 at *P* < 0.001. The mean r^2^ for all materials was 0.480 (*P* < 0.001), and the LD in G2 (0.668, ranging from 0.068 to 1.000) was significantly (*P* < 0.001) larger than that in G1 (0.291, ranging from 0.066 to 1.000) (*P* < 0.01).

Populations with high levels of outcrossing have relatively low LD [58]. Among outcrossing maize (*Zea mays* L.), Remington et al. [59] found lower levels of LD among 47 SSR loci (9.7 % of SSR pairs performing LD at *P* < 0.01), compared to LD data from an SSR survey of inbred lines of maize, which showed high levels of LD [60]. For switchgrass, LD data comparisons showed a trend towards higher LD in G2 (mean r^2^ = 0.668) including 42 genotypes all belonging to upland ecotypes, compared with G1 (mean r^2^ = 0.291), which contained 76 genotypes, including 7 lowland ecotypes.

## Method

### Plant material

A total of 134 switchgrass genotypes, representing most of the natural geographical distribution areas of switchgrass supplied by the Plant Genetic Resources Conservation Unit, Griffin, Georgia USA were used in this study. These included 7 lowland genotypes originating from 5 US states and 127 upland genotypes originating from Belgium and 15 US states (Table 3). The full accession data and information on switchgrass germplasm comes from ARS GRIN (http://www.ars-grin.gov/). The 134 genotypes, including one seedling from each accession, were grown and maintained in the experimental farm of the Sichuan Agricultural University during the 2012 growing season.

**Table 3:** The 134 switchgrass samples used for marker (ISSR, SCoT, and EST-SSR) genotyping

Code	Plant ID	Plant name	Ecotype	Origin	Code	Plant ID	Plant name	Ecotype	Origin
1	PI315723	BN-8358-62	LL	North Carolina, US	68	PI642244	70SG 057	UL	North Dakota, US
2	PI414065	BN-14668-65	LL	Arkansas, US	69	PI642245	70SG 058	UL	North Dakota, US
3	PI421521	KANLOW	LL	Kansas, US	70	PI642247	70SG 060	UL	North Dakota, US
4	PI421999	AM-314/MS-155	LL	Kansas, US	71	PI642248	70SG 061	UL	North Dakota, US
5	PI422006	ALAMO	LL	Texas, US	72	PI642249	70SG 062	UL	North Dakota, US
6	PI607837	TEM-SLC 01	LL	Texas, US	73	PI642250	70SG 063	UL	North Dakota, US
7	PI607838	TEM-SLC 02	LL	Texas, US	74	PI642251	70SG 064	UL	North Dakota, US
8	PI315724	BN-10860-61	UL	Kansas, US	75	PI642252	70SG 065	UL	North Dakota, US
9	PI315727	BN-11357-63	UL	North Carolina, US	76	PI642254	70SG 067	UL	North Dakota, US
10	PI414066	GRENVILLE	UL	New Mexico, US	77	PI642256	70SG 069	UL	North Dakota, US
11	PI414067	BN-8624-67	UL	North Carolina, US	78	PI642257	70SG 071	UL	North Dakota, US
12	PI414068	BN-18758-67	UL	Kansas, US	79	PI642259	70SG 073	UL	North Dakota, US
13	PI421138	Carthage	UL	North Carolina, US	80	PI642260	70SG 074	UL	North Dakota, US
14	PI421520	Blackwell	UL	Oklahoma,US	81	PI642261	70SG 075	UL	North Dakota, US
15	PI421901	MIAMI	UL	Florida, US	82	PI642262	70SG 076	UL	North Dakota, US
16	PI422001	STUART	UL	Florida, US	83	PI642264	70SG 078	UL	North Dakota, US
17	PI422003	PMT-785	UL	Florida, US	84	PI642265	70SG 079	UL	North Dakota, US
18	PI422016	-	UL	Florida, US	85	PI642267	70SG 081	UL	North Dakota, US
19	PI431575	KY1625	UL	Kentucky, US	86	PI642268	70SG 082	UL	North Dakota, US
20	PI442535	156	UL	Belgium	87	PI642269	71SG 001	UL	North Dakota, US
21	PI469228	Cave-in-Rock	UL	Illinois, US	88	PI642270	71SG 002	UL	North Dakota, US
22	PI476290	T2086	UL	North Carolina, US	89	PI642271	71SG 004	UL	North Dakota, US
23	PI476291	T2099	UL	Maryland, US	90	PI642272	71SG 005	UL	North Dakota, US
24	PI414069	BN-309-69	UL	New York, US	91	PI642275	71SG 008	UL	North Dakota, US
25	PI414070	BN-12323-69	UL	Kansas, US	92	PI642276	71SG 009	UL	North Dakota, US
26	PI476292	T2100	UL	Arkansas, US	93	PI642277	71SG 010	UL	North Dakota, US
27	PI476293	T2101	UL	New Jersey, US	94	PI642278	71SG 011	UL	North Dakota, US
28	PI476294	T4613	UL	Colorado, US	95	PI642279	71SG 012	UL	North Dakota, US
29	PI476295	T4614	UL	Colorado, US	96	PI642280	71SG 013	UL	North Dakota, US
30	PI476296	T16971	UL	Maryland, US	97	PI642281	71SG 014	UL	North Dakota, US
31	PI476297	Caddo	UL	Oklahoma,US	98	PI642282	71SG 015	UL	North Dakota, US
32	PI477003	Ncbraska 28	UL	Nebraska, US	99	PI642283	71SG 016	UL	North Dakota, US
33	PI478002	T6011	UL	North Dakota, US	100	PI642284	71SG 017	UL	North Dakota, US
34	PI537588	DACOTAH	UL	Oregon, US	101	PI642285	71SG 018	UL	North Dakota, US
35	PI549094	TRAILBLAZER	UL	Nebraska, US	102	PI642286	71SG 019	UL	North Dakota, US
36	PI591824	SHAWNEE	UL	Nebraska, US	103	PI642287	71SG 020	UL	North Dakota, US
37	PI598136	SUNBURST	UL	South Dakota, US	104	PI642288	71SG 021	UL	North Dakota, US
38	PI642190	FALCON	UL	New Mexico, US	105	PI642289	71SG 022	UL	North Dakota, US
39	PI642191	SUMMER	UL	South Dakota, US	106	PI642290	71SG 023	UL	North Dakota, US
40	PI642192	PATHFINDER	UL	Nebraska, US	107	PI642291	71SG 024	UL	North Dakota, US
41	PI642195	70SG 003	UL	North Dakota, US	108	PI642292	71SG 025	UL	North Dakota, US
42	PI642196	70SG 004	UL	North Dakota, US	109	PI642293	71SG 026	UL	North Dakota, US
43	PI642197	70SG 005	UL	North Dakota, US	110	PI642294	71SG 027	UL	North Dakota, US
44	PI642198	70SG 006	UL	North Dakota, US	111	PI642295	71SG 028	UL	North Dakota, US
45	PI642199	70SG 007	UL	North Dakota, US	112	PI642296	71SG 029	UL	North Dakota, US
46	PI642200	70SG 008	UL	North Dakota, US	113	PI642297	71SG 030	UL	North Dakota, US
47	PI642201	70SG 010	UL	North Dakota, US	114	PI642298	71SG 031	UL	North Dakota, US
48	PI642203	70SG 012	UL	North Dakota, US	115	PI642299	71SG 032	UL	North Dakota, US
49	PI642204	70SG 013	UL	North Dakota, US	116	PI642301	71SG 034	UL	North Dakota, US
50	PI642207	70SG 016	UL	North Dakota, US	117	PI642302	71SG 035	UL	North Dakota, US
51	PI642208	70SG 017	UL	North Dakota, US	118	PI642303	71SG 036	UL	North Dakota, US
52	PI642209	70SG 018	UL	North Dakota, US	119	PI642304	71SG 037	UL	North Dakota, US
53	PI642210	70SG 019	UL	North Dakota, US	120	PI642305	71SG 038	UL	North Dakota, US
54	PI642212	70SG 021	UL	North Dakota, US	121	PI642306	71SG 039	UL	North Dakota, US
55	PI642213	70SG 022	UL	North Dakota, US	122	PI642307	71SG 040	UL	North Dakota, US
56	PI642214	70SG 023	UL	North Dakota, US	123	PI642309	71SG 041B	UL	North Dakota, US
57	PI642217	70SG 026	UL	North Dakota, US	124	PI642310	71SG 042	UL	North Dakota, US
58	PI642218	70SG 028	UL	North Dakota, US	125	PI642311	71SG 043	UL	North Dakota, US
59	PI642229	70SG 041	UL	North Dakota, US	126	PI642312	71SG 044	UL	North Dakota, US
60	PI642232	70SG 044	UL	North Dakota, US	127	PI648366	70SG 053	UL	North Dakota, US
61	PI642233	70SG 045	UL	North Dakota, US	128	PI648367	70SG 070	UL	North Dakota, US
62	PI642234	70SG 046	UL	North Dakota, US	129	PI657660	Central lowa Germplasm	UL	Missouri, US
63	PI642235	70SG 047	UL	North Dakota, US	130	PI657661	Blackwell	UL	Kansas, US
64	PI642236	70SG 048	UL	North Dakota, US	131	PI657662	NEBRASKA28	UL	Nebraska, US
65	PI642237	70SG 049	UL	North Dakota, US	132	PI657663	Blackwell	UL	Kansas, US
66	PI642242	70SG 055	UL	North Dakota, US	133	PI657664	GRENVILLE	UL	New Mexico, US
67	PI642243	70SG 056	UL	North Dakota, US	134	PI659345	9086103	UL	New York, US

Note: “UL” refers to upland ecotype switchgrass, “LL” refers to lowland ecotype switchgrass

### DNA extraction and marker genotyping

Genomic DNA was extracted from tender leaves of each individual using a modified cetyltrimethylammonium bromide (CTAB) method [61]. ISSR [designed by the University of British Columbia (UBC set No. 9)], EST-SSR [62], and SCoT primer [45] sequences were aligned to the *Panicum* reference genome using the bl2seq blast program in NCBI (www.ncbi.nlm.nih.gov/BLAST/), which was designed to eliminate redundancies. Initially, four germplasms were used to screen marker primers [PI421999 (AM-314/MS-155), PI422006 (Alamo), PI642190 (Falcon), and PI642207 (70SG 016)]. The selected primers were synthesized by the Shanghai Sangon Biological Engineering Technology and Service Company (Shanghai, China) to genotype the collection.

ISSR-PCR was carried out according to Li et al [63] as follows: the total reaction volume was 15 μL and contained 20 ng template DNA, approximately 1.0 μM primer, 7.5 μL Mix (10 × PCR buffer, Mg^2+^, dNTPs; Tiangen Biotech, Beijing, China), and 1 U Taq polymerase. Amplifications were performed in a BioRad iCycle PCR machine (BIO-RAD Certified) under the following conditions: 95 °C for 5 min, followed by 35 cycles of the following: 95 °C for 45 s, 52–55 °C for 45 s, and 72 °C for 90 s. A final extension was conducted at 72 °C for 7 min. All PCR bands were visualized on 1 % polyacrylamide gel electrophoresis in 1 × TBE buffer. Silver staining was used to visualize the bands. The SCoT-PCR amplification reaction was conducted in a total volume of 15 μL according to Collard and Mackill [5], and containing 10 ng template DNA, 0.8 mM primers, 1.2 mM MgCl_2_, 0.4 mM dNTPs, and 1 U Taq DNA polymerase (Tiangen Biotech, Beijing, China). PCR amplification had an initial denaturation step of 5 min at 95 °C, followed by 45 s at 95 °C, 45 s at 55 °C, 1.5 min at 72 °C for 30 cycles, and 7 min at 72 °C. PCR products were visualized following agarose gel (1.5 %) electrophoresis at 120Vfor 1.5 h in 1 × TBE buffer, followed by staining with GelRed (Tiangen Biotech, Beijing, China). The EST-SSR PCR consisted of a denaturation for 5 min at 94 °C then 35 cycles of 30 s at 94 °C, 30 s at 53–55 °C, and 2 min at 72 °C, with a final extension of 5 min at 72 °C [62] and products were visualized as described above.

### Genetic variation and marker efficiency analysis

For each marker, polymorphic alleles were scored as “1” for presence and “0” for absence at the same mobility, and this data was used to construct an original data matrix. Using Excel 2007 and POPGENE v.1.32 [64], corresponding diversity parameters were estimated including: total number of bands (TNB), number of polymorphic bands (NPB), percentage of polymorphic bands (PPB), Nei’s (1973) gene diversity index (H), and Shannon’s information index (I). AMOVA v.1.55 was employed to reveal genetic variation among groups and within a population [65]. The data input to POPGENE and AMOVA was produced by DCFA v.1.1 [66].

The comparative efficiency of ISSRs, SCoTs, and EST-SSRs in these 134 switchgrass genotypes was assessed with MI. MI is the product of the EMR and the Ib_av_ for the polymorphic markers [67]. EMR is explained as the average number of polymorphic bands [68]. Ib_av_ is defined as:

1 $$ {\mathrm{Ib}}_{\mathrm{av}}=1/n{\displaystyle \sum }1-\left(2\mid 0.5-\mathrm{pi}\mid \right) $$

pi is the proportion of the i-th amplification site, n represents the total number of amplification site.

### Population structure analysis

The model-based program STRUCTURE v.2.3.4 (http://pritchardlab.stanford.edu/structure.html) [69] was applied to assess the population structure of the 134 switchgrass genotypes with 51 ISSRs, SCoTs, and EST-SSRs. The number of subpopulations (K) was set from 1 to 10 based on admixture models and correlated band frequencies. With 5 × 10^5^ Markov Chain Monte Carlo replications carried out for each run after a burn-in period of 10^6^ iterations, 20 independent runs were performed per K. When there was a clear maximum value for posterior probability [LnP(D)] output in STRUCTURE, a K value was selected in the range of 1 to 10 subpopulations. The most probable K value was the ΔK, an ad hoc quantity related to the rate of change in LnP(D) between successive K inferred by STRUCTURE [70]. The replication of K showing the maximum likelihood was applied to subdivide the genotypes into different groups with membership probabilities ≥ 0.75. Genotypes with less than 0.75 membership probabilities were assigned to an admixed group. Bar charts from the STRUCTURE data were displayed using Distruct 1.1 [71].

A dendrogram was drawn using FreeTree and TreeView programs (http://web.natur.cuni.cz/flegr/freetree.php) [72] based on Nei-Li genetic similarity coefficient with unweighted pair group method average (UPGMA) clustering.

To reveal relationships among the 134 switchgrass genotypes, a figure of two-dimensional scatterplots representing all of the genotypes was obtained for principal coordinate analysis (PCA) using NTsys-pc v.2.1 [73]. All of the switchgrass individuals were analyzed to calculate MRD [74]. The resulting genetic distance matrices were double-centered and used to obtain eigenvectors by the modules DCENTER and EIGEN using NTsys-pc.

### Evaluation of linkage disequilibrium

The significance of pairwise LD was evaluated using squared band-frequency correlations (r^2^) between all combinations of marker loci using the package TASSEL version 2.1 (http://www.maizegenetics.net/bioinformatics) [75]. Rare bands with a band frequency of less than 5 % were removed to avoid biased evaluations of LD because of their large variances. Other pairs of bands were evaluated with a minor band frequency of at least 5 % (MAF ≥ 0.05) with the GDA 1.1 program [76].

## Conclusions

The results of this study showed a great level of genetic variation among switchgrass germplasm. The switchgrass accessions were clearly divided into two groups containing upland and lowland ecotypes. For the first time, we revealed the extent of LD and population structure in switchgrass. The implications of these results in terms of utilizing association mapping for genes or QTL discovery in switchgrass were discussed. For further association mapping using a collection of switchgrass samples, we highly recommend the inclusion of more lowland ecotypes or the use of other nuclear markers in conjunction with ISSR, SCoT and EST-SSR.

## Abbreviations

CTAB: cetyltrimethylammonium bromide
EMR: effective multiplex ratio
EST-SSR: expressed sequence tag-simple sequence repeats marker
H: Nei’s gene diversity index
I: Shannon’s information index
Ib_av_: band informativeness
IRAP: inter-retrotransposon amplified polymorphism
ISSR: inter simple sequence repeat marker
LD: linkage disequilibrium
LL: lowland ecotypes
MI: marker index
MRD: modified Rogers distances
NPB: number of polymorphic bands
PCA: principal coordinate analyses
PPB: percentage of polymorphic bands
SCoT: start codon targeted marker
SSR: simple sequence repeat marker
TNB: total number of bands
UL: upland ecotypes
UPGMA: unweighted pair group method average
